# Cardiac Stem Cells in the Postnatal Heart: Lessons from Development

**DOI:** 10.1155/2018/1247857

**Published:** 2018-06-24

**Authors:** Cristina Aguilar-Sanchez, Melina Michael, Sari Pennings

**Affiliations:** Centre for Cardiovascular Sciences, Queen's Medical Research Institute, University of Edinburgh, Edinburgh EH16 4TJ, UK

## Abstract

Heart development in mammals is followed by a postnatal decline in cell proliferation and cell renewal from stem cell populations. A better understanding of the developmental changes in cardiac microenvironments occurring during heart maturation will be informative regarding the loss of adult regenerative potential. We reevaluate the adult heart's mitotic potential and the reported adult cardiac stem cell populations, as these are two topics of ongoing debate. The heart's early capacity for cell proliferation driven by progenitors and reciprocal signalling is demonstrated throughout development. The mature heart architecture and environment may be more restrictive on niches that can host progenitor cells. The engraftment issues observed in cardiac stem cell therapy trials using exogenous stem cells may indicate a lack of supporting stem cell niches, while tissue injury adds to a hostile microenvironment for transplanted cells. Engraftment may be improved by preconditioning the cultured stem cells and modulating the microenvironment to host these cells. These prospective areas of further research would benefit from a better understanding of cardiac progenitor interactions with their microenvironment throughout development and may lead to enhanced cardiac niche support for stem cell therapy engraftment.

## 1. Cell Turnover in the Heart: A Loss of Mitotic Potential

The heart has been a focus since the earliest medical research, yet some of the basic knowledge of heart cell biology has remained uncertain for almost a century. Before the concept of stem cells was known, a question was how the heart could maintain its essential function as a hard working organ throughout a human lifespan. A comparative lack of dividing cells had been observed in the adult heart by early histological detection of mitotic cells. Analyses of DNA synthesis in rodent heart tissues over subsequent decades indicated that the rate of DNA synthesis was extremely low in normal heart muscle and slightly increased in injured adult heart, whereas it was much higher during development and until adolescence [1]. Cardiomyocytes were found to stop dividing in the postnatal period when a switch occurs from hyperplasia to hypertrophy during terminal differentiation, and further heart growth is achieved through cell enlargement [2]. In rodents, this was detected by an increase in binucleated cells produced by cardiomyocytes synthesising DNA without completing cell division [3]. Human cardiomyocytes, which are less frequently arrested in a binucleated state (26–60%) than rodent cells (up to 90%), instead show increasing mononuclear polyploidy in the first decades of life [2–4]. Binucleated cells were speculated to provide metabolic benefit through increased transcription of mRNA [5], at the expense of cell renewal.

For many decades, it was taught that the heart was essentially restricted in cell number after birth, unable to regenerate after injury, and adapting to increased workload through cell enlargement. Studies using labelling and other techniques had nevertheless suggested some cardiomyocyte renewal; this was proposed to balance a rate of cell loss through apoptosis and called for a reevaluation of the terminally differentiated state of ventricular myocytes in the adult mammalian heart [6, 7]. The highest reported heart cell renewal rates raised the prospect of several tissue replacements per lifetime, as well as new cardiomyocyte generation after injury [8]. This led to a widening range of experimental data [9] and a useful revision of the dogma, but it was not easily understood in view of the clinical prevalence of heart failure, a chronic condition highlighting the lack of cardiac regenerative capacities. However, it was noted that organ damage including fibrosis is irreversible even in organs with high cell turnover, suggesting these are separate issues [6]. The field was more reconciled with studies using a method based on ^14^C isotope decay measurement in humans. This estimated the rate of cardiomyocyte DNA synthesis in adulthood as less than 1% per year, following a gradual decrease from childhood [4, 10]. It was calculated that less than half of cardiomyocytes may be replaced during a normal lifespan [10]. Interestingly, in adult heart, the cell renewal rates of endothelial cells (>15% per year) and mesenchymal cells (<4% per year) were much higher than those of cardiomyocytes [4]. The overall arrest in cell division of cardiomyocytes after birth in mammals is not as yet explained but is associated with downregulation of positive cell cycle regulators, as well as centrosome disassembly [3, 11]. The potential for cell division is thought more likely to be retained in mononucleated cells or in smaller cells [5]. In lower vertebrates, however, the mitotic apparatus seems preserved [11]. Zebrafish displays a higher regenerative potential of organs including the heart, where the response to injury was found to reactivate cardiomyocyte proliferation of a subset of cells undergoing limited dedifferentiation [12–14]. In mammals, a low rate of cardiovascular replacement was confirmed and traced back to existing dividing cardiomyocytes [15].

Following revision and debate, it was proposed that cell turnover in the mammalian heart muscle occurs at a very low rate [16], which may contribute to its structural maintenance. It is normally insufficient to heal the heart after injury and in disease, but conditions or drugs may be identified that can stimulate the cells retaining mitotic potential [10]. Such cells remain abundant in lower vertebrates, but in mammals, these cells are predicated on rare mitotic cardiomyocytes or on the existence of progenitor and stem cells in an adult cardiac niche. The key to understanding the fate of proliferating cells in the adult heart may be found during its development, when active cell division is supported in dynamic cardiac microenvironments.

## 2. Heart Development: Assembling Progenitor Cells from Different Sources

**Figure 1:**
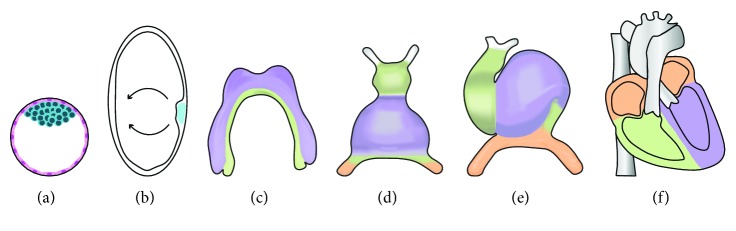
Summary of mouse heart development. (a) E3.5 preimplantation blastocyst stage showing pluripotent inner cell mass (ICM); (b) E6.5-gastrulating embryo showing mesoderm formation (arrows); (c) at E7.5, myocardial progenitor cells migrate to form the cardiac crescent; (d) at E8, the cardiac crescent fuses at the midline to form the early cardiac tube; (e) at E8.5, the cardiac tube forms a loop; (f) at E12.5–E15.5: the chambers undergo septation.

Heart development is marked by growth transitions producing a cardiac tube and then causing its looping and partitioning until it reaches its final full-sized chambered heart structure (Figure 1). The process starts soon after embryo gastrulation at embryonic day 6.5 (E6.5) of mouse development, when the mesoderm is formed between the ectoderm and the endoderm germ layer during ingression through the primitive streak. The earliest cardiac progenitors are bilateral groups of cells that originate in the anterior mesoderm and migrate and extend across the ventral midline into a cardiac crescent at E7.5, which is referred as the first or primary heart field [17]. They are joined by a second group of progenitor cells from the underlying pharyngeal mesoderm forming the second heart field [18]. By E8, cardiac crescent cells migrate to the midline, merging the abutments of this arch to form a primitive heart tube. This is composed of beating cardiomyocytes lined with endothelial endocardial cells, separated by an extracellular matrix (ECM) named cardiac jelly. Endocardial cell commitment is thought to occur prior to their migration into the heart field [19]. The slightly later differentiating cells from the second heart field add to the ends of the heart tube to become the arterial and venous poles [18]. The initial heartbeat is found at the inflow region of this heart tube, but subsequent pacemaker cells are thought to arise from the right lateral plate mesoderm [20, 21]. At this stage, the heart tube already functions as a valveless pump with a compound mechanism [22]. Looping and bulging of this rapidly growing tube creates the left ventricle from primary heart field cells, and most of the right ventricle and the outflow tract from secondary heart field cells. By E10.5, the venous poles have pushed up anteriorly and dorsally to form the future atria composed of cells from both fields. Development is completed with the septation of the chambers and valve formation from endocardial cushions by E15.5 [23]. During this time, cells of neural crest origin migrate from the dorsal neural tube and complete the separation of the outflow tracts. Progenitor cells from the extracardiac mesoderm, termed the third heart field, migrate to an anterior location on the heart where they form a transient structure, the proepicardium. Proepicardial cells generate the epicardium by gradually covering the heart towards its apex [24, 25]. Lineage tracing showed that proepicardial cells are also the source of coronary vasculature cells [19]. The niche created at the interface between epicardium, myocardium, and nascent vasculature recruits migrating fetal macrophages of yolk sac origin [26]. Cardiac fibroblasts are also thought to originate from cells migrating out of the proepicardial organ or from epithelial to mesenchymal transition (EMT) during valve formation [27]. Importantly, this suggests that the role of the early mesoderm progenitors in organogenesis is taken over by a proepicardial niche in later cardiac development [24].

## 3. Developmental Signalling Environments: Inducing Cell Proliferation and Differentiation

**Figure 2:**
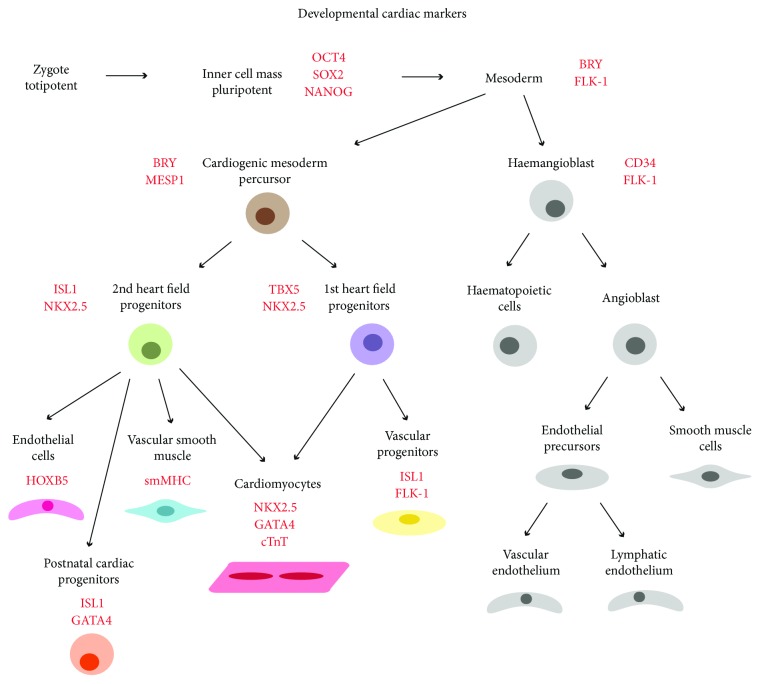
Summary of some of the cell differentiation stages with characteristic transcription factor markers (red) during embryonic development from zygote to cardiac and endothelial tissue.

Signalling from the surrounding microenvironment directs the transcription regulation of the developmental programme of the heart, necessary for differentiation (Figure 2) as well as proliferation. Specification of the cardiac progenitors is induced by endoderm-produced bone morphogenic protein (BMP) and suppressed by neural Wnt signals [28]. *Gata4* and NKX2.5 are the central transcription factors common to cardiac progenitors, whereas *Tbx5* and ISL1 are specific to cardiac progenitors of the first and second heart fields, respectively [29, 30]. These progenitors differentiate primarily into cardiomyocytes forming heart muscle but also endocardial cells forming the endothelial lining, as well as endothelial cells and vascular smooth muscle cells forming the blood vessels. Cardiomyocytes can further specialise into pacemaker cells generating the electrical impulses and the Purkinje cells conducting these [20, 21]. Other progenitors lead to the cardiac fibroblasts in connective tissues, the epicardial cells forming the outer layer of the heart, pericytes, and resident immune cells [23]. Signalling between these cells further determines morphogenesis in the developing heart [31]. For instance, during development, embryonic cardiac fibroblasts promote cardiomyocyte proliferation through ECM/*β*1 integrin signalling. In addition, endocardial release of neuregulin 1 (NRG-1) regulates cardiomyocyte differentiation and proliferation necessary for trabecular growth within the ventricles, along with NOTCH1, VEGFR-2, and FGF signalling [32]. In turn, the myocardium releases ANG-1 required for differentiation and proliferation of the endocardium [33]. Myocardium also releases TGF-*β*, BMP, Wnt, and Notch signals regulating the EMT of cells in the endocardium during valve development [31]. Conduction cells differentiate from a subset of contractile cardiomyocytes in response to paracrine signals including endothelin-1 [34]. Epicardial retinoic acid (RA) activates FGF signalling important for proliferation in compact myocardium and for inducing downstream Wnt signalling promoting EMT for growth of the coronary vasculature. In turn, signalling from the myocardium regulates epicardial development [31, 35].

The dependence on signalling pathways in heart development [28] shows that these provide proliferation and differentiation cues from the earliest specification of progenitors in the cardiac crescent to the final heart chamber formation. Niche interactions occur through soluble paracrine signals or physical contacts through integrins and cadherins, which are coupled with cytoplasmic receptors that transduce these signals to the nucleus, where they regulate transcription [36]. Additionally, heart morphogenesis is directed by mechanoregulation from the nascent circulation, pressure load, and myocardium contractility [37, 38]. These signals are transduced via various cell sensors that respond to flow, pressure, stretching, and rhythmicity [39]. The resulting differential gene expression patterns are supported and stably propagated through new cell lineages by epigenetic mechanisms [40]. Heart developmental gene regulation was shown to be determined by chromatin remodelling, histone acetylation and methylation, and DNA methylation [41–45]. The heart has not only provided an early example of the contributions of epigenetic modifiers of gene expression to organogenesis; interestingly, it showed a partial reactivation of developmental histone deacetylases in adult disease [46–49]. In addition, the chromatin-remodelling complex BRG1 was reported to reactivate in response to cardiac stress [50, 51]. However, outside a developmental environment, adult cardiomyocyte reactivation results in cell growth rather than proliferation [49]. Similarly, the fate of progenitors seems to be affected by the transition from developing tissues to the mature configuration of the adult heart.

## 4. The Adult Cardiac Microenvironment: Confining Space and Signals for Function

**Figure 3:**
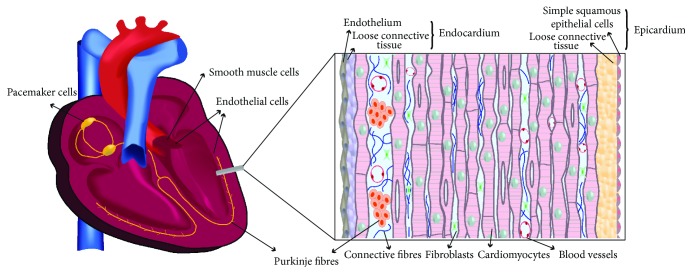
Adult heart architecture with left ventricle wall cross section showing the myocardium organisation with the endocardium lining and epicardium outer layers. Cell types drawn are mature cardiomyocytes, cardiac fibroblasts in their collagen matrix, endothelial cells of the endocardium and capillaries, Purkinje fibres, and epithelial and connective tissue cells of the epicardium.

Adult mammalian heart tissue has a specialised architecture that serves its essential contractile function (Figure 3). Cardiomyocytes are characterised by the ability of a subset of sinoatrial and atrioventricular nodal or Purkinje cells to generate action potentials and beat spontaneously; the automaticity of these cardiac pacemaker cells involves hyperpolarisation-activated and cyclic nucleotide-gated (HCN) channels [52, 53]. Contraction of cardiac muscle is produced by myofibrils formed by chains of sarcomeres, in which actin filaments interact with myosin filaments, the structural integrity of which is essential [54]. The left ventricular wall consists of lamellar units of myocardial cells in a helical arrangement, which gradually shifts in angle from a left-handed myocyte spiral in the outer zone, through a circumferential zone in the middle part, to a right-handed spiral in the inner zone of the wall [55]. In sections taken across the dense wall, these cardiomyocytes also show connections in radially twisted transmural sheets, which are less tightly coupled towards the inside wall of the left ventriculum [56].

A three-dimensional network of connective tissue surrounds and connects these myocardial sheets, lamellae, and cells, through an extracellular collagen matrix termed the perimysial weave [56]. This interstitial collagen is produced by cardiac fibroblasts, which are present in similar numbers as the cardiomyocytes in the adult heart [57, 58]. Cell-sorting measurements have shown that the proportion of fibroblasts is species specific, and that it is low in embryonic heart but increases during late foetal and neonatal growth [59], reflecting its longer period of proliferation. Nevertheless, the fibroblasts also stop dividing after heart maturation, although this is thought to be due to a quiescent state from which a subset can reenter the cell cycle [59]. Cardiac fibroblast markers such as discoidin domain receptor 2 (DDR2) and vimentin can distinguish these cells from cardiomyocytes expressing *α*-myosin heavy chain (*α*-MHC), cardiac troponin T (cTnT), HCN4, and NKX2.5 [60]. However, some cell markers are not found present in the whole population of cardiac fibroblasts or are not specific to this cell type alone [61]. This unusual fibroblast cell type can conduct electrical signals via connexins through gap-junctional coupling with each other as well as with cardiomyocytes [61, 62], showing it contributes structurally as well as functionally to heart function.

Other abundant occupants of the heart are the endothelial cells, which can be endocardial (the lining of the heart) or vascular (coronary arterial, venous, capillary, and lymphatic cells) [19]. Recent methodology suggests that endothelial cells are more numerous than the other main cell types, but they only make up a small volume [58]. A high density of capillaries in the myocardial interstitial space ensures the supply of oxygen and nutrients to other cells, as well as communication via paracrine factors released by endothelial cells including nitric oxide, reactive oxygen species, endothelin-1, natriuretic peptides, and cytokines [35]. The epicardium forms the outer layer of the adult heart composed of connective tissue, adipose tissue, and surrounding mesothelium, a single layer of epithelial cells in contact with the pericardial fluid [25]. In addition to coronary vessels and nerves, the subepicardium niche environment remains host to macrophages and several other cell types identified by electron microscopy, including immature cardiomyocytes [26, 63]. Several of these cell types have mesenchymal stem cell (MSC) characteristics or other markers of potential progenitor cells in the heart [64].

Overall, the dense construction of mature myocardium embedded in a fibroblast matrix with the endothelial capillary network, with signalling integral to cardiac physiology and its contractile function, leaves few potential sites for adult cardiac stem cell-supporting niches. The loose connective subepicardial tissue surrounding the heart remains a separate niche environment featuring mixed cell types including potential progenitors. These are known to differentiate into several cell types, including cardiomyocytes [65]. Interstitial spaces around blood vessels in the myocardium are further high nutrient environments for interactions between resident and itinerant cells. Microscopic evidence for adult stem cell niches was reported at such locations in the atria and apex [6, 66].

## 5. The Cardiac Microenvironment in Disease: Stress Signals and Responses

Cardiac fibroblasts can proliferate in response to pathological stimuli [57, 59]. The source of these activated cardiac fibroblasts was initially thought to include resident cells and circulating progenitors [57], rapidly infiltrating a site of injury. More recent lineage tracing studies suggest that the response involves mainly resident cardiac fibroblasts, although contributions from perivascular cells and epicardial cells are possible [61, 67, 68]. Abnormal ECM changes during injury cause activated cardiac fibroblasts to undergo a TGF-*β*-induced and mechanoregulated differentiation to myofibroblasts, which express *α*SMA, fibronectins, stress fibres, and contractile activity. This initial response to heart injury can eventually lead to cardiac remodelling and chronic heart failure [69]. Further unexpected roles of myofibroblasts have been suggested in regulating apoptotic engulfment [70].

Cell division of preexisting cardiomyocytes is low but is increased adjacent to areas of myocardial injury, whereas it is reduced by aerobic respiration-mediated oxidative DNA damage [15, 71]. Stress signalling in the myocardium furthermore switches on genes encoding fetal isoforms of proteins [72]. Normally quiescent epicardial cells also proliferate to form epicardium-derived cells (EPDCs) that differentiate into mesenchymal cells; whether these can subsequently populate the adult myocardium is under debate [27]. An important extrinsic factor altering the cardiac niche is the inflammatory response occurring after myocardial infarction. In the ischemic phase, the infarcted tissue suffers necrosis and release of cytokines triggered by tumor necrosis factor TNF-*α*. Reperfusion brings on a further damaging inflammation response with recruitment of neutrophils, monocytes, and further cytokines, which trigger fibroblast proliferation and neovascularization [73]. The gross changes following cardiac injury and inflammation lead to an increase in myofibroblasts that will initially repair, then remodel the heart with a more rigid ECM [74]. This maladaptive response overshadows the reactivation of other progenitors or proliferating cells in this overall nonregenerative environment.

## 6. Resident Cardiac Progenitors: Uncovering Residual Heart Developmental Capacity

The existence of progenitor populations in the adult heart has been the focus of many studies [75]. The criteria in the search for cardiac progenitor cells are that they should reside in the heart as a self-renewing pool of multipotent cells able to differentiate into the main cardiac lineages.  Table 1 summarises reported examples of cardiac resident side population cells, ISL1+ progenitors, c-Kit+ cells, Sca1+ cells, epicardial progenitors, and mesenchymal cells. Here, we will focus on the cells that have been investigated in more detail.

### 6.1. Cardiac c-Kit+ Cells

Multipotent, clonogenic, and self-renewing c-Kit+ cells isolated from bone marrow were first claimed to be able to substantially regenerate injured myocardial tissue [82]. Resident c-Kit+ cells in the heart were also reported to have this regenerative capacity [83, 84], suggesting a role in cardiac repair. In the heart, these cells were found together with supporting cells in niches that controlled the migration and differentiation of residing c-Kit+ cells [66]. However, other research groups reported that c-Kit+ cells in adults did not become cardiomyocytes [85, 86], or that they became cardiomyocytes through cell fusion with very low frequency [87], or that c-Kit+ cells could indeed generate new cardiomyocytes during ageing and after injury, but in extremely small quantities [88]. While this generated a debate regarding the reliability and sensitivity of the reporter mouse models [89, 90], it was demonstrated that an endothelial population of cells in mouse hearts expressed *c-Kit*, inconsistent with its role as a marker of uncommitted cells [91]. This result supported the finding that c-Kit+ cells are endothelial cells that are very infrequently capable of dedifferentiating to cardiac stem cells [88, 91]. More recent lineage tracing experiments confirmed a small subset of cardiomyocytes (∼0.03%) expressing *c-Kit* in the adult heart, in addition to more abundant c-Kit+ cardiac endothelial fates [92]. This study and others cautioned that lineage tracing is based on a binary readout potentially overestimating contributions from transient or low expression, as actual cardiac *c-Kit* expression levels in cardiac resident cell populations isolated from adult heart may be low. On the other hand, underestimating factors in c-Kit+ cell genetic fate-mapping studies may include inefficient Cre recombinase activity in cardiac stem cells and deleterious consequences of *c-Kit* haploinsufficiency resulting from genetic manipulation of the endogenous *c-Kit* gene locus. These technical limitations have contributed to the ongoing debate on rare adult stem cell populations, which confirmed the low abundance of cardiac stem cells among the c-Kit+ cardiac cells and their very low levels of *c-Kit* expression [93]. Nevertheless, c-Kit+ identifies cardiovascular progenitors during development capable of differentiating into the major cardiac lineages until at least the neonatal stage in mammals, so an important question is how their cardiac myogenic capacity is largely lost in the adult [85, 94].

### 6.2. Epicardial Progenitors

During development, epicardium-derived cells are known for their capability of undergoing epithelial-to-mesenchymal transition (EMT), invading the heart and differentiating into other cells, such as the cardiac fibroblasts [27]. A subset of Wilm's tumour 1 Wt1(+) mouse epicardial cells was reported to differentiate into cardiomyocytes and integrate into the myocardium [97]. Furthermore, a population of multipotent *Tbx18*-expressing proepicardial progenitors was reported to differentiate into cardiomyocytes, cardiac fibroblasts, and coronary smooth muscle cells [29]. These genetic lineage tracing studies relied on the epicardial specificity of these markers during development, and their findings were complicated by reports already detecting these markers in the myocardium [98, 99]. In adult mice, epicardium-derived progenitors residing in the epicardium and subepicardium were proposed to be resident adult cardiac stem cells. Stimulating the reexpression of the embryonic epicardial marker *Wt1* in these cells by priming with thymosin *β*4 peptide was reported to enhance their response to subsequent injury and promote differentiation to cardiomyocytes [65]. However, this reprogramming of epicardial to cardiomyocytes was not observed when thymosin *β*4 treatment was administered after myocardial injury, when this was tested as a treatment model [100]. The adult epicardium remains a niche for progenitors that undergo EMT upon myocardial infarction and migrate to the subepicardium, where they differentiate into myofibroblasts and smooth muscle cells [101].

### 6.3. Cardiac Mesenchymal Cells

MSCs are adult stem cells that can be isolated from many tissues and on this basis may be resident in the heart [102]. However, cardiac MSCs are not unambiguously distinguishable by specific markers or morphology, so they have been defined by their differences from other cells or grouped with related cell types based on shared markers [4, 64]. Furthermore, MSCs are defined by self-renewal and multipotency criteria following in vitro tissue culture, while their primary in situ properties in many organs are still under debate [103]. Adult human heart pericytes purified from myocardium express MSC/stromal cell markers, but their multipotency seems restricted [104]. Cardiomyocyte differentiation potential was reported to be limited in cardiac mesenchymal cells, whether these had been derived from myocardium or from subpericardium origins [101, 104]. In a myocardial infarction model, these cells contributed paracrine benefits but differentiated into mesenchymal cells, not cardiomyocyte or endothelial fates [101]. Adult cardiac-resident MSC-like stem cells with a proepicardial origin were described as colony-forming units-fibroblasts (CFU-Fs), which expressed platelet-derived growth factor receptor alpha *(Pdgfrα)* and *Sca1* [105]. These can give rise to many cell fates but mainly cardiac stromal/fibroblast cells.

The adult cardiac niche contains quiescent stem cells and progenitors; some of which can reactivate in response to injury, but current evidence suggests that these cells generate primarily noncardiomyocyte cell fates or contribute to the low rate of cardiomyocyte turnover. Adult heart progenitors that can differentiate into other cell types such as endothelial cells are regarded as beneficial for tissue maintenance and regeneration through their prosurvival and angiogenic functions [79]. In strategies aiming to repopulate the heart, exogenous stem cells such as MSC and reprogrammed cells have received considerable attention as an accessible and more abundant source of stem cells.

## 7. Reprogrammed Cardiomyocytes: Recreating Heart Developmental Potential

### 7.1. Cardiomyocytes from Pluripotent Stem Cells

**Figure 4:**
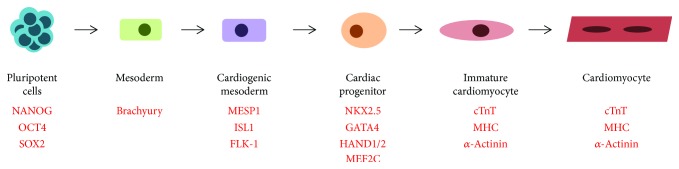
Diagram illustrating differentiation of pluripotent cells to cardiomyocytes. Markers for identification are shown for each step (red). ES or iPS cells differentiate towards mesoderm and cardiac mesoderm through cardiac progenitors and become mature, spontaneously contracting cardiomyocytes.

Among the cell replacement approaches towards cardiac regeneration is the use of cardiomyocytes obtained by in vitro derivation from embryonic stem (ES) or induced pluripotent stem (iPS) cells [106]. Similar to development, a pluripotent stem cell in a dish differentiates through the stages of mesoderm, cardiac mesoderm, and then cardiac progenitor before finally giving rise to a cardiomyocyte (Figure 4), with markers allowing identification of each differentiation stage [107]. ES and iPS cells begin to differentiate in culture by forming embryoid bodies [108, 109] when LIF is removed from the culture medium [110, 111]. The differentiation process is directed by the expression of transcription factors, recapitulating in vivo developmental stages of differentiation [110] (Figure 2). A cardiomyocyte-like fate also occurs directly in differentiation medium containing fetal calf serum, nonessential amino acids, and beta-mercaptoethanol [108, 112].

One of the issues of producing cardiomyocytes from fibroblast-derived iPS cells for cell therapeutic use is the heterogeneity of lineages, in which the cells will differentiate, resulting in a variety of cells aside from cardiomyocytes and raising tumourigenicity concerns. Inhibition or activation of specific signalling pathways, such as treatment with glycogen synthase kinase 3 inhibitors and then Wnt signalling inhibitors [113] or optimising the levels of the signalling molecule BMP4, key players in the specification of cardiac mesoderm, improved the efficiency of cardiomyocyte induction from iPS cells [28, 114]. Another issue is that cardiac lineage cells mature during the differentiation process in vivo while their beating frequency and sarcomere organisation increases. However, cardiomyocyte cultures obtained from ES or iPS cells consist mostly of immature cells with varying levels of sarcomeric organisation and inconsistent spontaneous contraction [115, 116]. Coculturing of cardiomyocytes differentiated from ES cells with endothelial cells was reported to improve their maturity and upregulate several microRNAs, which when transfected could replicate the effect [117]. This shows that developmental microenvironments can assist in directing pluripotent stem cells to form cardiac progenitors and cardiomyocytes in vivo, as well as in vitro.

### 7.2. Cardiomyocytes Transdifferentiated from Somatic Cells

Adult cardiomyocytes can also be obtained by derivation of other somatic cells, such as fibroblasts [75, 106]. Cell reprogramming combines a dedifferentiation of fibroblasts to induced pluripotent stem (iPS) cells with directed differentiation to cardiomyocytes. Transdifferentiation is a technique in which differentiated cells are reprogrammed to different cell lineages by direct conversion, without going through a pluripotent stage. This has permitted the production of, for example, neurons [118], cardiomyocytes [119], or endothelial cells [120]. A clinical advantage of transdifferentiation of somatic cells is that they could be taken from the patient, thus reducing the danger of rejection, although such treatment may not be cost-effective and there is some debate regarding the immunogenicity of reprogrammed cells [121, 122].

It has been shown that transdifferentiation reprogramming occurs without passing through an ISL1+ cardiac progenitor cell stage [119]. These cells show activation of genes that are specific for cardiomyocytes, such as ryanodine receptor 2 *(Ryr2)*, connexin43 *(Gja1)*, cTnT, and *α*-MHC [119]. Three cardiac development transcription factors, *Gata4*, *Mef2c*, and *Tbx5*, are sufficient for cardiomyocyte induction in vitro and were also used to reprogramme cardiac fibroblasts to cardiomyocytes in mice in vivo after myocardial infarction, reducing scar tissue [123]. Fibroblast conversion to cardiomyocytes could be increased several-fold by adding *Hand2* to the transcription factor cocktail [124] or by enhancing *Mef2c* expression relative to *Gata4* and *Tbx5* [125]. Through further refinement, cardiomyocyte reprogramming was achieved to specific cardiac cells such as Purkinje [126] or pacemaker cells [127]. An alternative transdifferentiation protocol used three iPS factors, *Oct4*, *Klf4*, and *Sox2*, to initiate reprogramming and then direct cardiomyogenesis by adding BMP4 and inhibiting Janus kinase (JAK1) [128, 129].

## 8. Stem Cell Engraftment in the Cardiac Niche: Stem Cell Therapies Needing Microenvironments

The use of exogenous allogeneic stem cell injection strategies has focused on various populations: bone marrow-derived cells including MSCs, progenitor cell populations, pluripotent cells, and in vitro differentiated cells. Early reports suggested that injected stem cells were not detectably retained inside cardiac niches, but that positive effects were nevertheless observed as a result of paracrine signalling by these cells. A meta-analysis of stem cell therapy in the mouse model demonstrated a significant improvement in left ventricular ejection fraction [130]. The first reported animal studies detected engrafted cells [131, 132]; however, very low engraftment rates following intramyocardial cell injection are common. Myocardial engraftment in the rat model was improved using a prosurvival cocktail with Matrigel [133], suggesting that the allogeneic stem cell microenvironment can be modulated.

A large number of clinical trials have since demonstrated a good safety record for injecting stem cells into patients after myocardial infarction. Comprehensive surveys of these trials concluded an overall modest efficacy in improving cardiac function, indicating considerable variance and discrepancy with animal studies, while the long-term benefits remained uncertain [134–138]. For instance, of a dozen studies using injected bone marrow mononuclear cells, three noted an improved left ventricular ejection fraction, whereas other studies did not record differences in the patients' cardiac parameters, and the optimal timing of these injections after cardiac injury also remained unclear [135]. The much larger BAMI randomised controlled trial may be more conclusive on bone marrow mononuclear cells [134]. On the other hand, MSCs are immune-privileged, permitting the use of allogeneic transplants, and they are also a better characterised homogeneous population [106]. Smaller studies evaluating allogeneic bone marrow MSC injections were generally encouraging [139–141]. Overall, these and other studies indicate that the benefits of cardiac cell therapy may depend on more purified cell populations or better reprogrammed cells, while there is no consensus regarding the best cell type materials, which are still evolving [134].

In addition, cardiac stem cell therapies have much to gain from improved cell engraftment. Cell retention, long-term engraftment, and cell survival have been ongoing issues, with an estimated 1% of donor cells surviving after 4 weeks. Low engraftment is due to initial washout and thereafter the hostile inflammatory environment of the injured tissue, immune rejection, or the lack of mechanical resistance of the donor cells [134]. It is also possible that these stem cells undergo epigenetic changes in culture [142, 143]. Strategies towards enhancing cell survival by preconditioning the cells for the microenvironment of the transplantation site have been encouraging, as well as modulation of the microenvironment at the injured cardiac site [133, 134]. This shows that further investigation of potential adult cardiac niches and a better understanding of the processes by which developmental progenitors are supported by their cardiac microenvironment could lead to more effective cardiac stem cell therapies.

## Figures and Tables

**Table 1:** Cardiac progenitor cells and their activity in the heart.

Cardiac resident progenitor type	Characteristics	Cardiac cell fate contribution	References
Side population cells	Perivascular cells of undetermined origin; can grow as cardiospheres	Embryonic heart: cardiomyocytes, endothelial cells	[76–78]
		Adult: endothelial cells	

ISL1+ cardiac progenitors	Major population of undifferentiated cardiac progenitors during development	Embryonic heart: cardiomyocytes	[79–81]
		Adult: cardiomyocytes (rare)	

c-Kit+ cells	Cardiovascular progenitors during development, may be confined to endothelial fate or localised to niches in adult	Embryonic heart: endothelial cells, cardiomyocytes	[66, 82–94]
		Adult: endothelial cells, cardiomyocytes (rare)	

Sca-1+ cells	Heart resident endothelial cells sharing characteristics with mesenchymal cells and side population cells	Embryonic heart: mesenchymal, endothelial, other	[81, 95, 96]
		Adult: cardiomyocytes (low-level replacement)	

Epicardial progenitors	Capable of epithelial-to-mesenchymal transition, multipotent progenitor potential during development, may be reinducible in adult	Embryonic heart: cardiomyocytes, cardiac fibroblasts, coronary smooth muscle cells	[27, 29, 65, 97–101]
		Adult: myofibroblasts and smooth muscle cells, cardiomyocytes upon induction	

Mesenchymal/stromal cells	Expressing MSC/stromal cell markers, restricted multipotency compared to other MSC	Adult heart: mainly cardiac stromal/fibroblast cells, limited cardiomyocyte potential	[4, 64, 102–105]
